# Validation of a Turkish Translation of the Stress in Emergency Healthcare Professionals: The Stress Factors and Manifestations Scale

**DOI:** 10.1002/nop2.70780

**Published:** 2026-08-30

**Authors:** Halil Alsancak, Fatma Birgili

**Affiliations:** ^1^ Department of Nursing Muğla Health Sciences Faculty, Muğla Sıtkı Koçman University Muğla Türkiye

**Keywords:** emergency health care professionals, reliability, scale, stress factors, stress symptoms, validity

## Abstract

**Aim:**

The primary duties of emergency healthcare professionals (EHPs) are to provide emergency patient care to acutely ill and injured individuals. Due to the nature of their work, EHPs operate under constant stress, often requiring rapid decision‐making, swift action, and the delivery of necessary medical care in life‐or‐death situations, sometimes under inadequately safe conditions. Therefore, the aim of this study is to determine the validity and reliability of the Emergency Healthcare Professional Stress Factors and Symptoms (SEHP:SFMS) Scale in Turkish for identifying stress factors and symptoms in emergency medical care professionals providing emergency patient care services.

**Design:**

A methodological study design was used in this study.

**Methods:**

The study was conducted with the participation of 211 EHPs from employees working in emergency care institutions affiliated with the Muğla Provincial Health Directorate between November 2023 and June 2024. Data were collected via a face‐to‐face survey. Data were analysed using Lawshe content validity ratio, Kaiser–Meyer–Olkin coefficient, Bartlett test, exploratory factor analysis, principal component analysis, Varimax factor rotation method, confirmatory factor analysis, Cronbach's α internal consistency coefficient, convergent validity, discriminant validity, test–retest, and Spearman correlation coefficient tests.

**Results:**

The linguistic translation and cultural adaptation of the SEHP:SFMS showed strong performance. The scope validity index of the scale is 0.83. The item‐total correlation values of the scale were found to be between 0.486 and 0.794, and the factor loadings were between 0.474 and 0.816. Confirmatory factor analysis fit indices: χ^2^ = 248.727; df = 101; *n* = 211; *p* = 0.000; χ^2^/df = 2.463; RMSEA = 0.083; CFI = 0.914, SRMR = 0.052, which was found to be compatible and acceptable with the proposed 3‐factor model. The Cronbach's α reliability coefficient of the scale was 0.931, and the total variance was 61.97%.

**Conclusions:**

SEHP:SFMS is a valid and reliable tool to assess stress factors and symptoms of Turkish emergency healthcare professionals. Its use improves the quality of emergency care.

**Patient or Public Contribution:**

These study findings have been used to create a tool with Turkish validity and reliability that allows for the examination of stress factors among healthcare professionals working in emergency and critical services. Identifying and reducing stress factors among healthcare professionals is crucial for the delivery of quality healthcare services. It can also be used to develop targeted interventions and ongoing strategies to facilitate improved clinical supervision and mentoring.

**Implication for Nursing Practice:**

Nurses in emergency departments, which are among the most stressful, dynamic, intense, life‐saving, and critical environments in healthcare institutions, and where life‐saving treatment is administered, are at high risk of experiencing psychological trauma. Trauma experienced in the work environment is a significant problem for nursing. The consequences of trauma negatively affect nurses and institutions. Studies show that post‐traumatic stress, anxiety, depression, and burnout are commonly observed in emergency department nurses. In this sense, understanding the stress and stress factors experienced by nurses can guide future interventions. The results of this study are considered important in making visible the stress and stress factors experienced by nurses in the emergency department, and also in guiding managers and nurses working in this field in terms of preventive and protective measures.

## 1 Introduction

Hospitals are businesses that provide uninterrupted diagnostic and treatment services 24 h a day (Özdaş and Kızılkaya 2021) and are generally considered one of the most stressful work environments (Kendrick et al. 2020). Emergency departments in hospitals are also units that provide continuous service, where occupational accidents and risks are most frequent, and mortality rates are high. Therefore, due to the intense emotions such as uncertainty, anxiety, and fear experienced by patients and their relatives who apply to emergency departments, and the frequent occurrence of violent incidents, emergency departments are considered the most stressful units of hospitals (Saleh et al. 2020; Bingöl and İnce 2021). For these reasons, emergency department personnel experience higher levels of stress compared to employees in other units due to the continuous and intense nature of their work, their responsibilities, and other stress factors (Kendrick et al. 2020; Ferkai et al. 2024). Stress is defined as a dynamic process in which individuals adapt to or exhibit maladaptive responses to perceived threats or dangers arising from changes in the internal or external environment (Liao et al. 2023). Prolonged and intense occupational stress can lead to sequential outcomes, such as increased job dissatisfaction (Afshari et al. 2021; James et al. 2023), adverse health consequences (Liao et al. 2023), burnout (Søvold et al. 2021), poor sleep quality (Khan et al. 2020), and even chronic diseases (McCormick et al. 2023; Snyder et al. 2020). These manifestations may include physical symptoms (e.g., headaches, hypertension, and cardiovascular diseases), psychological symptoms (e.g., irritability, restlessness, anguish, anxiety, withdrawal, resistance, depression, occupational burnout syndrome, fear, and worry), cognitive impairments (e.g., forgetfulness and attention deficits) (Liao et al. 2023; Afshari et al. 2021), or occupational hazards such as injuries and infections (Bikmaz 2020).

Emergency healthcare professionals (EHPs) are expected to deliver exceptional performance in life‐threatening situations (Ferkai et al. 2024). This challenging work environment exposes them to constant acute stress, leading to deficiencies in the performance of complex cognitive tasks. It can even lead to medical errors during the delivery of multiple healthcare services, jeopardising patient health and safety (Ferkai et al. 2024; Sedlár 2022). Furthermore, these adversities can negatively impact the optimism of both healthcare workers and patients (Sedlár 2022), as there is an inverse relationship between optimism and stress (McCormick et al. 2023; García‐Tudela et al. 2022). EHPs commonly perform their duties under high stress (James et al. 2023; Taylor et al. 2023). Given these challenges, there is a need for validated tools to assess stress levels among these professionals. Such tools can facilitate the development of strategies to improve job performance and quality of life while reducing errors caused by attention deficits. Although various instruments exist to measure stressors and symptoms, most of them focus on a single domain (Ippolito et al. 2025; Nilsson et al. 2020; Taylor et al. 2023). This scale, whose validity and reliability have been established in Turkish, comprehensively examines the life events, work‐related events, and known symptoms of stress experienced by emergency medical professionals in Türkiye. Additionally, the “Stress in Emergency Healthcare Professionals: The Stress Factors and Manifestations Scale (SEHP:SFMS)” is expected to assist researchers in more accurately measuring stress among both hospital‐based and pre‐hospital EHPs across diverse groups (García‐Tudela et al. 2022).

Therefore, the aim of this study is to translate SEHP:SFMS into Turkish for use in identifying stress factors and factors causing stress in the work environment among EHPs, and to determine its validity and reliability in Turkish. SEHP:SFMS will contribute to creating a theoretical framework that can guide future improvement practices for safe practice among EHPs.

## 2 Methods

### 2.1 Study Design and Procedure

The study is a methodological study. This study tested the validity and reliability of the Turkish version of the SEHP:SFMS, which was originally in English. The STROBE checklist was used to report the research context, study design, methods and results of the study.

### 2.2 Conceptual Framework and Item Generation

A questionnaire consisting of a total of 38 questions, including the EHP information form (17 questions) and SEHP:SFMS (21 items), was used to collect data.

The personal information form: This form, developed by the researchers based on the literatüre (García‐Tudela et al. 2022; Kelly 2020; Yin et al. 2020), included 17 questions on demographics (e.g., age, gender, marital status), workplace characteristics (e.g., unit, years of experience), stress factors, burnout experiences and sources, and turnover intention.

Stress in Emergency Healthcare Professionals: The Stress Factors and Manifestations Scale (SEHP:SFMS); The original SEHP:SFMS, developed in English by García‐Tudela et al. (2022) in Spain, consists of 21 items across four factors: Self‐concept (eight items), socialisation (six items), somatisation (four items), and illness symptoms (three items). The scale is a 5‐point Likert type (1: I completely disagree–5: I completely agree). The highest possible score on the scale was 105, and the lowest score was 21. The SEHP:SFMS Cronbach's α coefficients were found to be 0.908, 0.892, 0.810, 0.757, and 0.734, respectively (García‐Tudela et al. 2022). In our study, the Cronbach's α of SEHP:SFMS and its sub‐dimensions were calculated as 0.931, 0.907, 0.801, and 0.681, respectively.

### 2.3 Language Validity of SEHP:SFMS

Language validity was ensured through forward‐backward translation, following Beaton et al.'s (2000) guidelines (Beaton et al. 2000). The Davis technique (Evcimen and Bilgin 2024) was applied to evaluate linguistic equivalence, and a scoring scale was used to confirm language validity (Gürbüz 2021). The literal forward translation of the SEHP:SFMS from its source language (English) into the target language (Turkish) was completed by five independent translators. The five initial translated versions were evaluated by the researchers with advisors, and the most appropriate expressions were merged into a single draft after achieving consensus. Turkish language and literature were checked by a linguistic lecturer in terms of clarity of expressions and spelling. To determine the comprehensibility of the Turkish scale items, which were created after translation and content validity testing, a pilot study was conducted with 20 EHPs that met the acceptance criteria. The aim of the pilot study was to evaluate the comprehensibility, linguistic appropriateness, and applicability of the item statements in the Turkish form of the scale. Since no feedback was received regarding the scale items after the pilot study, the form created for the research was used. The data obtained from the pilot study were not included in the main sample and were not used in statistical analyses.

### 2.4 Content Validity of SEHP:SFMS

The Lawshe technique was used to assess the content validity of the study. This is a technique in which experts evaluate the relevance of each item of a measurement tool to the relevant topic or domain (Romero Jeldres et al. 2023). Two rounds of content validity assessment were conducted to determine whether the assessment was representative of emergency department professionals, including both emergency medicine specialists and nursing academics. A total of 10 experts, including ten emergency medicine academics, five nurse academics, and three final‐year EHPs, were consulted to provide their opinions on the content validity of the items. Ten experts were asked to rate the language translation accuracy of 16 translated items on a 1 to 4‐point scale using the SEHP:SFMS, where (1) Not appropriate, (2) somewhat appropriate (item/phrase revision needed), (3) quite appropriate (appropriate but minor changes needed), and (4) extremely appropriate. The scores obtained from the experts were as follows: 8/10 experts (80%) received 3–4 points, and 3/10 (30%) received 2 points. The Content Validity Index (CVI) was calculated by dividing the number of experts who received 3–4 points from the items by the total number of evaluations. Necessary corrections were made to the survey using the expert comments. Then, the content validity ratio CVI for each item and the CVI for the overall scale were calculated (the CVI of the survey was 0.8) (Lawshe 1975). According to the Lawshe technique, the CVI of the scale items for 10 experts should be at least 0.42 at the α = 0.05 significance level (Ayre and Scally 2014).

### 2.5 Participants

The study population comprised 1014 EHPs, including registered nurses, emergency medicine specialists, general practitioners, paramedics, and emergency medical technicians working in hospitals and ambulance teams, under the Muğla Provincial Health Directorate. However, the fundamental approach in scale adaptation studies is not to reach the entire population, but rather to ensure a sufficient sample size for psychometric analyses. In scale adaptation, it is necessary to reach at least 5–10 times as many people as the number of questions in the scale (Akgül 2005). For this reason, a minimum of 210 EHPs, which is 10 times the 21‐item SEHP:SFMS, was targeted. First, using the Simple Random Sampling method, a sample frame containing all elements of the population was determined. Researchers assigned a number to each element within the sample frame. Then, researchers generated as many random numbers as the number of elements they wanted to include in the sample and randomly selected them from the list (Kılbaş and Cevahir 2023). The study sample consisted of 211 EHPs, including 111 registered nurses The study sample consisted of 111 registered nurses (Muğla Training and Research Hospital (17), Yatağan State Hospital (9), Menteşe State Hospital (8), Milas State Hospital (14), Bodrum State Hospital (12), Kavaklıdere State Hospital (6), Marmaris State Hospital (9), Datça State Hospital (4), Köyceğiz State Hospital (6), Ortaca State Hospital (7), Dalaman State Hospital (4), Fethiye State Hospital (11), and Seydikemer State Hospital (4)); eight emergency medicine specialists (Muğla Training and Research Hospital (6), Yatağan State Hospital (1), and Fethiye State Hospital (1)), and 25 general practitioners (Muğla Training and Research Hospital (4), Yatağan State Hospital (4), and Menteşe State Hospital (2)); 37 paramedics (working in ambulance teams under the Muğla Provincial Ambulance Service Chief Physician's Office, affiliated with the Muğla Provincial Health Directorate); 30 emergency medical technicians (working in ambulance teams affiliated with the Muğla Provincial Ambulance Service Chief Physician's Office, which is under the Muğla Provincial Health Directorate). Participants were selected using a random sampling method. The study included 211 participants in total, representing approximately 21% of the total. Due to the voluntary nature of participation, demanding working conditions, shift work systems, and the high workload of emergency medical services, it was not possible to reach the entire population. The intense and irregular working conditions of healthcare professionals, particularly those working in emergency departments and ambulance units, limited the data collection process. Furthermore, detailed data on individuals who did not participate in the study were unavailable, making it impossible to evaluate their characteristics. Therefore, the possibility of selection bias cannot be completely ruled out. However, the sample size obtained is considered sufficient for psychometric analysis, and the inclusion of emergency medical workers from different professional groups is considered to provide sufficient diversity for the scale's construct validity.

### 2.6 Data Collection

The study data were collected from emergency medical professionals working in the emergency departments of hospitals affiliated with the Muğla Provincial Health Directorate and in ambulance teams affiliated with the Muğla Provincial Ambulance Service Chief Physician's Office between November 2023 and June 2024. A 21‐item questionnaire containing participant characteristics (7 questions) and SEHP:SFMS items was used for data collection. Data were collected through face‐to‐face interviews by four researchers responsible for data collection. After obtaining approval from their clinical supervisors, the researchers informed the participating emergency medical professionals about the study and asked those who agreed to participate to sign a written consent form. The data collection form was explained to the participants in detail, and they were asked to complete the forms, which took 25–30 min. The completed forms were collected from the students in sealed envelopes by the researchers. A test–retest analysis was conducted for the reliability analysis of the draft scale (Strenier et al. 2024). For this purpose, 97 randomly selected emergency medical professionals were asked to complete the scale again after 2 weeks. The CVI value was calculated as 0.83.

### 2.7 Data Analysis

Descriptive statistics, including frequencies, percentages, and mean ± standard deviation values, were calculated for all variables. Item analysis was conducted to evaluate the compatibility of items with the scale. This involved item‐total correlations, Pearson correlation analysis, and exploratory factor analysis (EFA) (Büyüköztürk 2020). Items with correlations below 0.30 were removed from the scale because they both reduced the reliability of the scale and were not compatible with the scale (Ahmed and Ishtıaq 2021). The adequacy of the sample size was determined by a Kaiser–Meyer–Olkin (KMO) value > 0.6, and Bartlett's test of sphericity was *p* < 0.05 (de et al. 2021). In EFA, the Principal Component Principal Axis Factor Analysis (PAFA) method and Varimax Rotation Technique were applied to calculate factor loadings. Eigenvalues were used to determine the number of factors (Karagöz 2021). To test the accuracy of the created measurement model and EFA results, EFA and CFA were performed using the same dataset (*n* = 278). Acceptable values for fit indices for CFA analysis: χ^2^/df value below 5, Root Mean Square Error of Approximation (RMSEA), Standardised Root Mean Square Residual (SRMR) values below 00.08 (Büyüköztürk 2020) and Comparative Fit Index (CFI) values above 00.85 (Karagöz 2021). Cronbach's α (0.70) and item‐total correlations (≥ 0.30) are acceptable values for the internal consistency and reliability of the Turkish version of the SEHP:SFMS (Bolarinwa 2015; Eliüşük 2018; Polit and Beck 2020). Test–retest analysis was performed on 97 EHPs at three‐week intervals to determine the stability of the scale over time. In the analysis, ICC stands for Intraclass Correlation Coefficient. Test–retest reliability was evaluated using a two‐way mixed‐effects model, Consistency, Single Measures [ICC(3.1)]. The classification suggested by Koo and Li (2016) was used to interpret the ICC values (< 0.50 = weak, 0.50–0.75 = moderate, 0.75–0.90 = good, > 0.90 = excellent reliability). *p* < 0.05 was considered statistically significant. SPSS Version 26.0 and AMOS Version 24 package programs were used for statistical analysis of all data. In the entire study, analyses were performed by considering significance levels of 00.05 and 00.01 (de et al. 2021). In this study, data entries were checked, and it was determined that there were no missing responses in the dataset of 211 participants. Furthermore, prior to analysis, the normality of the data distribution was evaluated by examining skewness and kurtosis values. The obtained values were found to be within acceptable limits, and the data were accepted as conforming to a normal distribution.

### 2.8 Ethical Considerations

Ethical permission was obtained from the Ethics Committee of a University (date: 23.07.2023, protocol no: 230082, decision no: 97). Written institutional permission was obtained for conducting this research (E‐15682851‐770‐234,336,200). In addition, permission was received from García‐Tudela via email for the scale used in the validity and reliability study. All participants were informed about the purpose of the study before data collection, and they were assured that the data would be kept confidential and used only for scientific purposes. Additionally, participants were asked to sign a written informed consent form. This study was conducted in accordance with the principles of the Declaration of Helsinki.

## 3 Results

### 3.1 Characteristics of EHPs

Of the EHPs who participated in the study, 65.9% were female, the average age was 29.85 ± 7.02, 70.6% worked in the emergency department, 52.6% were registered nurses, and the average number of years working in the emergency department was 4.99 ± 5.19. Of the EHP, 73% experienced burnout, 81.5% willingly chose their profession, and 35.5% wanted to leave their jobs, making up a significant percentage (Table 1).

**TABLE 1:** Demographic characteristics of the participants.

Demographic characteristics	*n*	%
Gender		
Male	72	34.1
Female	139	65.9
Age		
20–25 age	57	27.0
26–30 age	87	41.2
31 age and ↑	67	31.8
X̄ ± Ss (Min. –Max.)	29.85 ± 7.02 (20–54)	
Unit of work		
Emergency Room	149	70.6
Emergency Ambulance	62	29.4
Occupation		
Registered Nurse	111	52.6
Emergency Medicine Specialist	8	3.8
General Practitioner	25	11.8
Paramedic	37	17.5
Emergency Medical Technician	30	14.2
Years of work in emergency Department/Emergency ambulance		
1–5 years	148	70.1
6–10 years	26	12.3
11 years and ↑	37	17.5
X̄ ± Ss (Min. –Max.)	4.99 ± 5.19 (1–26)	
Experiencing burnout		
Yes	154	73.0
No	57	27.0
Wanting to quit		
Yes	75	35.5
No	136	64.5
Wanting to choose a profession		
Yes	172	81.5
No	39	18.5
Total	211	100

*Note:* f: frequency, Max.: maximum, Min.: minimum, Ss.: standard deviation, X̄: mean.

A total of 66.8% of EHPs working in the emergency department stated that they experienced stress factors. The most frequently reported stress factors were workplace stress (11.8%), working long hours (10%), being called to frequent shifts (8.9%), work chaos (9.5%), and staff shortages (9.2%). 30.3% of emergency ambulance workers experienced stress factors; the most frequently reported stress factors are management attitude (11.5%), workplace stress (11.2%), working long hours (9.4%), work chaos (10.4%), conflict in the workplace (9%) and staff shortage (9%) (Table 2). These stress factors encountered by EHPs provide important clues for improving work conditions and management processes.

**TABLE 2:** Data on stress factors in the emergency department and emergency ambulance.

Stress factors	*n*	%
The presence of a stress factor in the emergency department		
Yes	141	66.8
No	70	33.2
Stress factors in the emergency department^a^		
Frequent call‐ins	75	8.9
Long hours of work	84	10.0
Workplace stress	99	11.8
Workplace conflict	63	7.5
Work chaos	80	9.5
Conceptual confusion in the workplace	45	5.4
Attitude of management	68	8.1
Shortage of materials	44	5.2
Shortage of personnel	77	9.2
Fear of being called to work while on leave	39	4.6
Inequality	44	5.2
Inexperience	36	4.3
Number of patients per person	61	7.3
Inability to express oneself in the workplace	21	2.5
Total	840	100.0
Presence of stress factors in emergency ambulance		
Yes	64	30.3
No	147	69.7
Stress factors in emergency ambulance^a^		
Frequent call‐ins	22	7.9
Long hours of work	26	9.4
Workplace stress	31	11.2
Workplace conflict	25	9.0
Work chaos	29	10.4
Conceptual confusion in the workplace	18	6.5
Attitude of management	32	11.5
Shortage of materials	8	2.9
Shortage of personnel	25	9.0
Fear of being called to work while on leave	18	6.5
Inequality	15	5.4
Inexperience	10	3.6
Number of patients per person	11	4.0
Inability to express oneself in the workplace	5	1.8
Others	3	1.1
Total	278	100.0

^a^ Multiple‐choice questions.

Table 3 reveals a statistically significant difference (*p* < 0.05) in the scores for general stress factors and symptoms, psychological stress, behavioural stress, and somatic stress among emergency healthcare workers based on their burnout status. According to these data, emergency healthcare workers experiencing burnout have higher scores for general stress factors and symptoms, psychological stress, behavioural stress, and somatic stress compared to those not experiencing burnout. Variables such as “burnout”, “stress factors” and “intention to leave the job” were assessed through single‐item, multiple‐choice questions prepared by the researchers in line with the literature (Birgili et al. 2024).

**TABLE 3:** Comparison of SEHP:SFMS scores according to EHPs’ burnout status.

Scale and subdimension	Burnout status	*n*	X̄	Ss	t	*p*
Scale Total	Yes	154	2.62	0.82	6.69	0.000**
	No	57	1.85	0.50		
Psychological stress	Yes	154	3.07	0.95	7.92	0.000**
	No	57	2.00	0.63		
Behavioural stress	Yes	154	2.76	0.92	6.11	0.000**
	No	57	1.95	0.66		
Somatic stress	Yes	154	2.03	0.95	3.27	0.001**
	No	57	1.59	0.56		

*Note:* X̄: Mean, Ss.: Standard deviation, *p < 0.05, **p < 0.01, t: Independent Sample *t*‐test.

### 3.2 Construct Validity of SEHP:SFMS

In this study, EFA and CFA were performed using the same dataset (*n* = 278).

#### 3.2.1 Exploratory Factor Analysis (EFA)

There were 21 items in the original form of the SEHP:SFMS. In the analysis of the evaluations made by the experts on the 21 items, it was seen that there was harmony between the opinions of the experts (*χ* ^2^(21) = 71.85, *p* < 0.01). In the calculation of factor loadings in the EFA, the Principal Axis Factoring Analysis method and Varimax Rotation Technique were applied. It was requested that the factor loading values be above 00.30 (Evcimen and Bilgin 2024). As a result of the consecutive factor analysis, items 7, 9, 10, 14, and 17 were removed from the study. As a result of EFA, the KMO value was00.929. The result of Bartlett's test was found to be a chi‐square value (*χ* ^2^ = 1777.26), degrees of freedom (df = 120), and *p* = 00.000.

#### 3.2.2 Confirmatory Factor Analysis (CFA)

**FIGURE 1:**
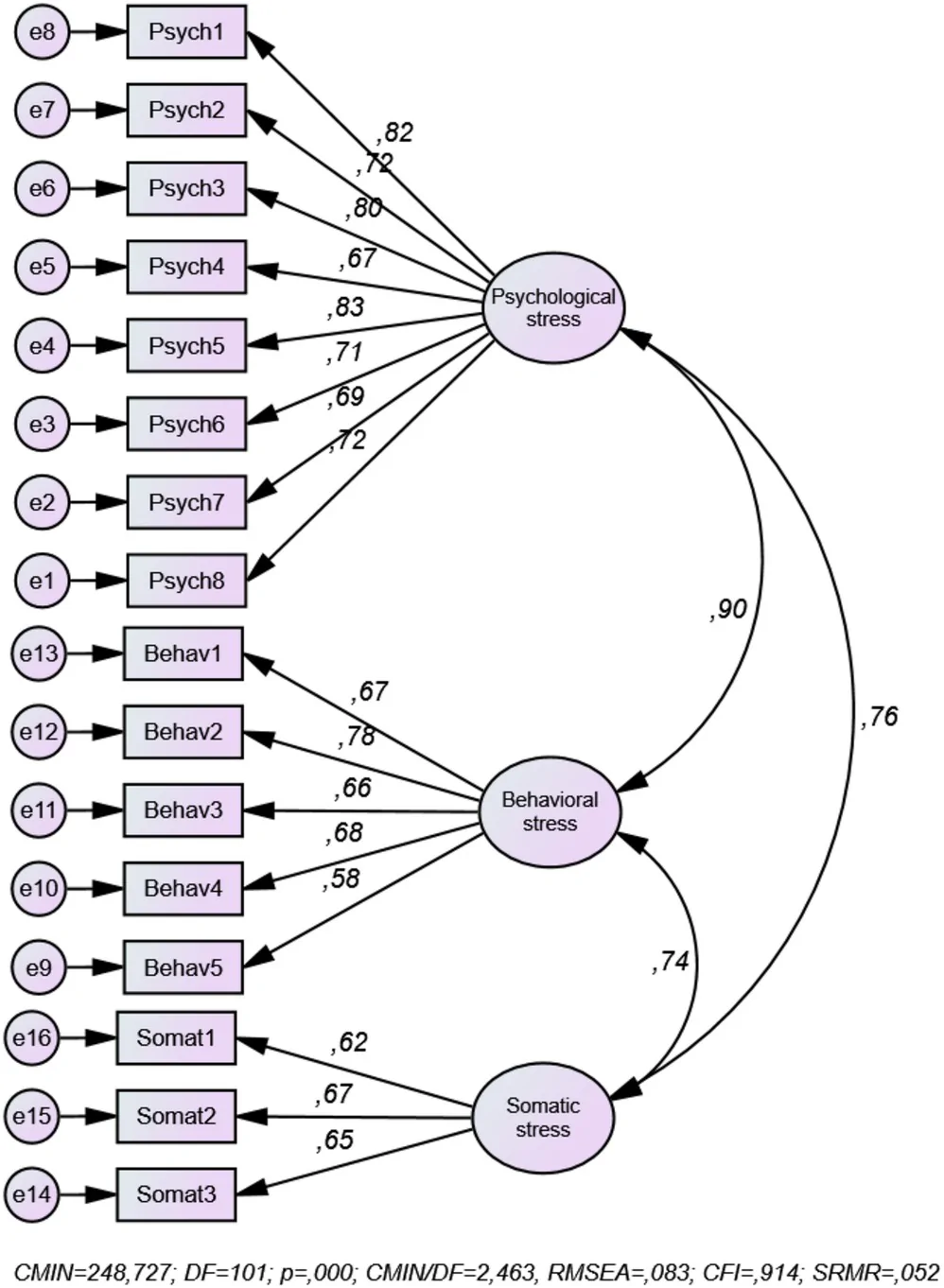
CFA of the three‐factor model. *s: Item.

The goodness‐of‐fit values (*χ* ^2^ = 248.727; df = 101; *N* = 211; *p* = 00.000; *χ* ^2^/df = 2.463; RMSEA = 00.083; CFI = 00.914; and SRMR = 00.052) of the SEHP:SFMS CFA result indicate that the proposed 3‐factor model is consistent with the data and is borderline or acceptable (Haradhan 2018). The parameter values of the CFA results of the SEHP:SFMS are shown in Figure 1.

SEHP:SFMS's eigenvalues, variances, and factor analysis results were examined; after rotation, the eigenvalue of the first factor, called “Psychological Stress”, is 4.42, and the factor explains 27.65% of the total variance. The eigenvalue of the second factor, called “Behavioural Stress”, is 3.03; the factor explains 18.96% of the total variance. The eigenvalue of the third factor, called “Somatic Stress”, is 2.46; the factor explains 15.36% of the total variance. The total variance is 61.97% (Table 4).

**TABLE 4:** Eigenvalues and variances of the scale before and after rotation.

Factor	Before rotation After rotation			Before rotation After rotation		
	Core values	Variance (%)	Cumulative variance (%)	Core values	Variance (%)	Cumulative variance (%)
1	7.74	48.37	48.37	4.42	27.65	27.65
2	1.15	7.21	55.58	3.03	18.96	46.61
3	1.02	6.40	61.97	2.46	15.36	61.97

### 3.3 Internal Consistency

In this study, Cronbach's α coefficients of the SEHP:SFMS and its subdimensions were calculated as00.931,00.907,00.801, and 0.681, respectively. The item factor loadings of SEHP:SFMS varied between 00.517–00.816 for factor 1, 00.474–00.723 for factor 2, and 00.607–00.774 for factor 3. It was seen that “the item‐total correlation for factor 1 varied between 00.644–00.794”, “the item‐total correlation for factor 2 varied between 00.542–00.662”, and “the item‐total correlation for factor 3 varied between 00.486–00.517” (Table 5). All correlation coefficients were found to be > 0.3. These results showed that the Turkish version of the SEHP:SFMS had satisfactory internal consistency and reliability.

**TABLE 5:** Results of item factor loadings, reliability (cronbach α) and item‐total correlation analysis of the scale.

Factors and items belonging to factors	Factor loadings	Item‐total correlation	Cronbach α
Psychological Stress			0.907
I feel restless	0.769	0.779	
I feel anxious	0.816	0.690	
I feel disgusted	0.656	0.747	
I sleep worse than usual	0.605	0.634	
I feel overwhelmed	0.726	0.797	
I put things off until later	0.685	0.681	
I have muscle pain or tension	0.517	0.644	
I often have doubts	0.567	0.661	
Behavioural Stress			0.801
I have a negative attitude towards others	0.723	0.611	
I feel aggressive	0.699	0.662	
I am not healthy enough to go to work	0.474	0.561	
I tend not to trust people	0.584	0.560	
I have digestive problems	0.660	0.542	
Somatic Stress			0.681
I feel sweating in some parts of my body	0.713	0.486	
I feel itching in some parts of my body	0.607	0.488	
I have heart palpitations	0.774	0.517	
SEHP:SFMS			0.931

Abbreviation: SEHP:SFMS, Stress in Emergency Healthcare Professionals: The Stress Factors and Manifestations Scale.

### 3.4 Test–Retest Reliability

A high level of positive correlation was observed between the SEHP:SFMS scores of the EHPs at two different times (*p* < 0.05) (Table 6).

**TABLE 6:** Relationship between SEHP:SFMS in EHPs applied at different times.

Variables	Coefficient
1. Application	*r* = 0.807**
2. Application	*p* = 0.000

*Note:* *p < 0.05, **p < 0.01.

Abbreviation: SEHP:SFMS, Stress in Emergency Healthcare Professionals: The Stress Factors and Manifestations Scale.

According to these data, the level of agreement between the two scale scores was high (Table 7).

**TABLE 7:** Test–retest reliability and temporal agreement results of the SEHP:SFMS scale and its subdimensions.

Scale/Subdimension	ICC	%95 Confidence interval	*p*
Scale total	0.807	0.714–0.872	< 0.001*
Psychological stress	0.878	0.817–0.918	< 0.001*
Behavioural stress	0.716	0.576–0.810	< 0.001*
Somatic stress	0.714	0.573–0.809	< 0.001*

*Note:* Test–retest reliability was assessed using a two‐way mixed‐effects model, Consistency, Single Measures [ICC(3,1)]. The classification suggested by Koo and Li (2016) was used to interpret the ICC values (< 0.50 = weak, 0.50–0.75 = moderate, 0.75–0.90 = good, > 0.90 = excellent reliability).

Abbreviation: ICC: Intraclass Correlation Coefficient.

* *P* < 0.05: It was considered statistically significant.

The scale was administered to 97 EHPs at two different times, three weeks apart, and a test–retest analysis was conducted to evaluate the scale's consistency over time. The overall ICC value for the scale was determined to be 0.807, indicating a high level of temporal agreement. When the sub‐dimensions were examined, the Psychological Stress sub‐dimension showed a high level of agreement (ICC = 0.878), while the Behavioural Stress (ICC = 0.716) and Somatic Stress (ICC = 0.714) sub‐dimensions showed good temporal consistency. All ICC values were found to be statistically significant (*p* < 0.001), indicating that the scale provides stable and reliable measurements over time.

## 4 Discussion

Emergency services are high‐stress environments for patients and healthcare professionals. While conditions such as high patient traffic, working at night and on weekends, and not being able to use mealtimes during treatment and care services put a physical burden on the EHPs, constantly witnessing patients who cannot be treated and lives that cannot be saved puts a mental burden on healthcare professionals (Özdaş and Kızılkaya 2021). For this reason, this study aimed to create a Turkish version of the SEHP:SFMS, a tool that can evaluate life events, work events, stress factors, and known symptoms of stress as a whole in pre‐hospital settings.

The average age of the EHPs participating in the study was 29.85 years, the average number of years working in the emergency department was 4.99 years, and about three‐quarters of them experienced burnout, with 35.5% wanting to quit their jobs. In the original study, the average age of the EHP was 41.5 years, the average number of years working in the emergency department was 10.08 years, and 32.7% reported experiencing stress (García‐Tudela et al. 2022). These features are consistent with our findings.

The literature emphasises the need for a standard translation process for translations of measurement instruments in different languages, not only for linguistic compatibility but also for cultural compatibility (Eliüşük 2018; Evcimen and Bilgin 2024). Therefore, for the language validity of the scale, the first five experts translated the items. Then, for the content validity (CV) of the scale, 10 experts were consulted, and for the back‐translation, two experts were consulted (Beaton et al. 2000; Polit and Beck 2020). The content validity index (CVI) value of 21 items was calculated as00.83; this value above 00.80 shows that the scale had a high CVI (Ahmed and Ishtıaq 2021).

The most commonly used method to determine construct validity is factor analysis (FA) (Akgül 2005; Karagöz 2021). To decide whether to apply FA to the study data, the KMO value was first calculated, and the Bartlett test was performed. KMO is a coefficient with a value between 0 to 1 (Karagöz 2021), calculated to determine whether the sample size is sufficient for FA (Bolarinwa 2015). The Bartlett test is a method for evaluating the relationship between items in a scale (Bolarinwa 2015). In this study, these findings show that the sample size for FA is “perfect” and the correlation between the items is appropriate (Orçan 2018). Also, these results are consistent with the original scale.

EFA and DFA are two frequently used FA methods (Orçan 2018). In this study, EFA and CFA were performed using the same dataset (*n* = 278). While this approach is sometimes preferred in scale adaptation studies due to limited sample sizes, it is known that performing CFA on an independent sample provides stronger confirmation of structural validity. For construct validity of the scale, an EFA was first performed, and three factors with eigenvalues greater than one were determined; it was determined that 61.97% of the total variance was explained. A high explained variance indicates that the concept/construct is well‐measured (Gürbüz 2021). It is possible to say that the Turkish version of the SEHP:SFMS consists of three factors and measures three different concepts (Büyüköztürk 2020). In this study, we investigated whether the factor structure of the Spanish and Turkish versions of the SEHP:SFMS was confirmed through CFA (Özel and Kutlu 2024). Standardised regression coefficients ranged from 00.486 to 00.794; a positive relationship was found between all factors. Correlations of 00.7 and below indicate medium,0.7–0.9 correlations indicate high, and correlations above0.9 indicate a very high level of relationship (García‐Tudela et al. 2022; Öner and Erden 2024).

In the EFA, the factor loading values were required to be at least 00.30 (Evcimen and Bilgin 2024). In this study, items 7, 9, 10, 14, and 17 were removed from the scale not only due to “cultural differences,” but primarily because of psychometrically inappropriate factor loadings. During EFA, it was determined that these items had significant loadings under more than one factor, i.e., exhibiting cross‐loading. The difference between the factor loadings of these items was found to be quite low, remaining well below approximately 0.10 in some items. Psychometrically, an item having similar loadings under more than one factor indicates that the item does not clearly represent a particular structure. This situation can weaken the conceptual integrity of the scale and the interpretability of the factor structure. Therefore, these items were removed from the analysis to achieve a clearer, more consistent, and more distinctive factor structure. The three‐factor structure obtained after the removal of the items yielded both more stable results and a stronger distinction between factors. Therefore, the removal of items is based not only on the assumption of cultural incompatibility, but also directly on the results of the psychometric assessment and the aim of strengthening the construct validity of the scale. The Turkish version of the scale consists of 16 items and 3 sub‐dimensions. The sub‐dimensions were psychological stress (items 1, 2, 5, 8, 16, 18, 19, and 21), behavioural stress (items 4, 6, 11, 12, and 13), and somatic stress (items 3, 15, and 20). The original scale consists of 21 items and 4 sub‐dimensions: “Self‐concept (items 1, 2, 5, 6, 9, 10, 14, 16, 18, and 21),” “socialisation (items 4, 5, 7, 8, 12, 17, and 21),” “somatisation (items 8, 13, 19, and 20),” “disease symptoms (items 3, 11, and 15)” (García‐Tudela et al. 2022). The scale items and sub‐dimensions of the original and Turkish versions matched with different structures. A structure determined for one culture may appear in different dimensions in another (Eliüşük 2018). In this context, it is thought that the reason for the distribution of items across different sub‐dimensions is due to geographical and cultural factors (Eliüşük 2018; Çapık et al. 2018). Furthermore, changes were made to the factor structure to evaluate the psychometric suitability of the scale in the Turkish sample. In our study, the four‐factor structure of the original scale was examined first. However, the results of the EFA showed that the item distributions differed significantly from those in the original study. Specifically, it was determined that some items loaded under different factors and that the original four‐factor structure could not be consistently validated in the Turkish sample. Furthermore, the fact that items 5, 8, and 21 in the original scale were placed under more than one factor indicates an overlap in the factor structure. To evaluate this situation in more detail, CFA was performed on the original four‐factor structure. Although some goodness‐of‐fit indices were at an acceptable level, the interfactor correlation values were found to be quite high. In particular, correlation values above 00.90 indicate a significant level of overlap between factors and suggest that structures that should theoretically be different may be measuring similar concepts in practice. In psychometric evaluations, interfactor correlations approaching or exceeding 1 are considered a significant problem in terms of discriminant validity. Therefore, instead of forcibly preserving the original structure of the scale, a new EFA was performed to determine the most suitable and psychometrically stronger structure for our target population. The analysis resulted in a more stable, interpretable, and culturally more appropriate structure consisting of 16 items and three factors. This approach is thought to strengthen the construct validity and reliability of the Turkish version of the scale. Items 7, 9, 10, 14, and 17 were removed from the scale not solely due to “cultural differences,” but primarily because of psychometrically inappropriate factor loadings.

It is very important for researchers to carefully determine reliability and validity issues in scale studies (Ahmed I, Ishtıaq, 2021). It is desirable that the scales developed or adapted cross‐culturally are reliable. Internal consistency (item statistics, splitting the test in half) and invariance over time (test–retest method) were preferred for the reliability method of the scale (Bolarinwa 2015). Reliability, which determines whether the scale can measure all aspects, is internal consistency (Karagöz 2021). The most appropriate way to determine the internal consistency of the scale is to use Cronbach's α coefficient (Kula Kartal and Mor Dirlik 2016). The Cronbach's α coefficients of the original SEHP:SFMS and its sub‐dimensions were00.908,00.892,00.810,00.757, and 0.734, respectively (García‐Tudela et al. 2022). Cronbach α coefficients of the SEHP:SFMS and its sub‐dimensions adapted to Turkish were00.931,00.907,00.801, and00.681, respectively. From this, it is possible to say that the items are consistent with each other and measure the same concept, and that the internal consistency of the scale is high (Haradhan 2018). The difference between the distribution of the items in the sub‐dimensions in Spain and Turkey is reflected in Cronbach's α values. In our study, the Cronbach's α coefficient for the Somatic Stress subscale was calculated as α = 0.681. However, this subscale consists of only three items. It is known that Cronbach's α coefficient is a reliability coefficient affected by the number of items, and it is expected that the α coefficient will be relatively lower in subscales with a small number of items. Furthermore, according to the classification proposed by Karagöz (2021), Cronbach's α coefficients between 0.60–0.80 are considered quite reliable, and values between 0.80–1.00 are considered highly reliable. Therefore, the α = 0.681 value for the Somatic Stress subscale is both within the limits accepted in the literature and shows sufficient internal consistency considering the limited number of items in the subscale.

SEHP:SFMS factor loadings were found to be between 00.474–00.816; item correlations were between 00.486–00.794; all item correlations were > 0.3; the items were associated with the total scale and factors; and were statistically significant at the *p* < 0.05 significance level. All goodness‐of‐fit measures related to CFA were found to be within acceptable and high fit limits (Özel and Kutlu 2024). It is possible to say that the Spanish version of the scale has also been validated for the Turkish language. A high correlation coefficient was found between psychological stress and behavioural stress factors (*r* = 0.90). This can be considered as discriminant validity. However, since psychological and behavioural symptoms of stress in emergency healthcare workers are interconnected and often occur together, this high correlation was considered a theoretically expected finding. The simultaneous occurrence of psychological stress symptoms and behavioural manifestations, especially in healthcare professionals working under intense and continuous stress, can lead to a strong relationship between the two factors. Furthermore, the results of the EFA showed that items clustered significantly under different factors, cross‐loading was at an acceptable level, and each factor conceptually represented a different dimension. The results of the CFA also showed that each item represented the relevant latent variable significantly and adequately. Therefore, despite the high correlation between the factors, when the factor loadings, contents, and theoretical structures of the items are evaluated together, it is concluded that the sub‐dimensions of Psychological Stress and Behavioural Stress do not measure the same construct, but are two separate dimensions that complement each other but represent different aspects of stress.

Stress can positively or negatively affect not only physical and psychological health, but also individuals' daily attitudes andbehaviours. Stress should not always be considered a negative factor for the organism. While stressful conditions can hinder an individual's capacity, they can also enable them to realise it. Mild levels of stress can be stimulating, activating, and motivating for the individual. However, as the level of stress increases, psychological, physical, and behavioural problems can occur for the individual (Rowshan 2000). Hans Selye used the concept of stress to encompass both aspects: Good stress (eustress) and bad stress (distress). According to Selye (1976), an optimal level of stress is necessary for the organism to behave, work, and develop. In other words, beneficial stress is a motivation consisting of the sum of psychological and physical strengths required to overcome obstacles and solve problems. According to Baltaş (2021), stress, whether positive or negative, can be observed at every stage of an organism's life. Stress is necessary for personal development and satisfaction. In our study, emergency medical professionals who reported experiencing burnout had significantly higher total scores and all subscale scores on the SEHP:SFMS (Table 7). Specifically, individuals experiencing burnout had significantly higher levels of psychological stress, behavioural stress, and somatic stress compared to those not experiencing burnout (*p* < 0.01). These findings indicate a strong relationship between stress symptoms and burnout. Furthermore, the fact that 35.5% of participants stated they were considering leaving their jobs supports the idea that stress and burnout in emergency medical professionals can have significant consequences for professional sustainability. The results obtained are considered consistent with studies in the literature showing a relationship between stress, burnout, and intention to leave the job (Alzoubi et al. 2024; Kelly et al. 2021; Norful et al. 2023).

Accordingly, it is considered that the RMSEA value should not be evaluated in isolation; when considered together with other goodness‐of‐fit indices, the model generally shows acceptable fit. In our study, the RMSEA value was found to be 0.083. Under normal conditions, RMSEA values are usually reported with two decimal places, which corresponds to approximately the 0.08 threshold. Furthermore, the literature suggests different acceptance ranges for RMSEA rather than a single, definite threshold. Some sources, primarily Gürbüz (2021), state that RMSEA values between 00.08 and00.10, while indicating weak or borderline fit, can be considered acceptable. In addition, the fact that other goodness‐of‐fit indices (CFI = 0.914; SRMR = 0.052; χ^2^/df = 2.463) were at acceptable levels in our study supports the idea that the model generally shows sufficient fit.

Since a scale similar to SEHP:SFMS is not available in our country, stability analysis could not be performed using a parallel or alternative form with the test–retest method (Haradhan 2018). In this study, the test–retest technique was preferred in examining the invariance over time, and a correlation analysis was performed. In our study, the test–retest scale total score correlation value was00.807, and a statistically significant relationship was found (Bolarinwa 2015). In the original study, the internal consistency of the items according to Cronbach's α coefficient in the reliability of the scale was 00.911 in the test and 00.915 in the retest. The correlation and significant relationship show that both scales provide similar values in repeated measurements (test–retest reliability); therefore, it is a fairly consistent scale (Bolarinwa 2015). The scale was administered to 97 emergency medical professionals at two different times, two weeks apart, and a test–retest analysis was conducted to evaluate the scale's consistency over time. The overall ICC value for the scale was determined to be00.807, indicating a high level of temporal agreement. All ICC values were found to be statistically significant (*p* < 0.001), indicating that the scale provides stable and reliable measurements over time.

### 4.1 Implications for Nursing Practice

Emergency department nurses witness numerous traumatic events, including the resuscitation or death of infants, children, or young people, maintaining care and resuscitation for critically ill patients, organ loss, and sudden deaths. Nurses in emergency departments, which are among the most stressful, dynamic, intense, life‐saving, and critically involved in emergency situations requiring immediate intervention, are at high risk of experiencing psychological trauma. Trauma experienced in the work environment is a significant problem for nursing. The consequences of trauma negatively affect both nurses and institutions. Studies show that post‐traumatic stress, anxiety, depression, and burnout are common among emergency department nurses. Understanding the types of stress and stress factors experienced by nurses can guide future interventions. The results of this study are considered important for making the stress and stress factors experienced by nurses in the emergency department visible using the Turkish SEHP:SFMS, and for guiding preventive and protective measures for nurses, nursing managers, educators, or emergency department leaders working in this field.

### 4.2 Limitations

The study has some limitations. Firstly, this study was conducted with health professionals providing emergency and critical services at institutions affiliated with the health directorate in a province in western Türkiye. Secondly, it was not possible to reach the entire population due to the voluntary nature of participation, intense working conditions, shift work system, and the high workload of emergency medical services. The intense and irregular working conditions of healthcare professionals, especially those working in emergency departments and ambulance units, limited the data collection process. Thirdly, since detailed data on individuals who did not participate in the study were not available, the characteristics of those who did not participate could not be evaluated. Therefore, possible selection bias cannot be completely ruled out. However, it is thought that the sample size obtained is sufficient for psychometric analyses and that the scale provides sufficient diversity in terms of construct validity due to the inclusion of emergency medical workers from different professional groups. Fourthly, due to the limited sample size in our study, EFA and CFA were performed on the same dataset. Performing EFA and CFA on the same dataset can be considered a methodological limitation; therefore, future CFA studies using independent samples may be needed. The resulting scale can also be used for health professionals working in other provinces.

## 5 Conclusion

The SEHP:SFMS's language suitability, semantic equivalence in Turkish society, and content validity for comprehensibility were provided. The sample size of this study was sufficient for EFA. The original (21 items with four sub‐dimensions) and the Turkish adaptation of the SEHP:SFMS (16 items with three sub‐dimensions) created a structure that was different from the original factor structure. CFA fit indices were found to be acceptable. A high level of positive relationship was observed between the scores of the scale applied to the EHP at two different times, and the fit levels were high. All item correlations were > 0.3, and the total scale and factors of the items were at the *p* < 0.05 significance level. The scale obtained as a result of the Turkish adaptation study of the SEHP:SFMS is a practical, valid, and reliable measurement tool. Methodological limitations of the study include the fact that EFA and CFA were applied to the same dataset, and it is suggested that the factor structure of the scale should be validated in independent and larger samples in the future.

## Author Contributions

**Fatma Birgili:** conceptualization, investigation, writing – original draft, methodology, validation, visualization, writing – review and editing, software, formal analysis, project administration, data curation, supervision, resources. **Halil Alsancak:** conceptualization, investigation, writing – original draft, methodology, validation, resources, writing – review and editing, software.

## Funding

The authors have nothing to report.

## Ethics Statement

Ethical permission was obtained from the Ethics Committee of a University (date: 23.07.2023, protocol no: 230082, decision no: 97). Written institutional permission was obtained for conducting this research (E‐15682851‐770‐234,336,200). Furthermore, the Turkish validation results of the scale were also sent to the author, and their approval was obtained. This study was conducted in accordance with the principles of the Declaration of Helsinki.

## Conflicts of Interest

The authors declare no conflicts of interest.

## Data Availability

The data that support the findings of this study are available on request from the corresponding author. The data are not publicly available due to privacy or ethical restrictions.
